# A paucity of heterochromatin at functional human neocentromeres

**DOI:** 10.1186/1756-8935-3-6

**Published:** 2010-03-08

**Authors:** Alicia Alonso, Dan Hasson, Fanny Cheung, Peter E Warburton

**Affiliations:** 1 Department of Genetics and Genomic Sciences, Mount Sinai School of Medicine, New York, NY 10029, USA

## Abstract

**Background:**

Centromeres are responsible for the proper segregation of replicated chromatids during cell division. Neocentromeres are fully functional ectopic human centromeres that form on low-copy DNA sequences and permit analysis of centromere structure in relation to the underlying DNA sequence. Such structural analysis is not possible at endogenous centromeres because of the large amounts of repetitive alpha satellite DNA present.

**Results:**

High-resolution chromatin immunoprecipitation (ChIP) on CHIP (microarray) analysis of three independent neocentromeres from chromosome 13q revealed that each neocentromere contained ~100 kb of centromere protein (CENP)-A in a two-domain organization. Additional CENP-A domains were observed in the vicinity of neocentromeres, coinciding with CpG islands at the 5' end of genes. Analysis of histone H3 dimethylated at lysine 4 (H3K4me2) revealed small domains at each neocentromere. However, these domains of H3K4me2 were also found in the equivalent non-neocentric chromosomes. A surprisingly minimal (~15 kb) heterochromatin domain was observed at one of the neocentromeres, which formed in an unusual transposon-free region distal to the CENP-A domains. Another neocentromere showed a distinct absence of nearby significant domains of heterochromatin. A subtle defect in centromere cohesion detected at these neocentromeres may be due to the paucity of heterochromatin domains.

**Conclusions:**

This high-resolution mapping suggests that H3K4me2 does not seem sufficiently abundant to play a structural role at neocentromeres, as proposed for endogenous centromeres. Large domains of heterochromatin also do not appear necessary for centromere function. Thus, this study provides important insight into the structural requirements of human centromere function.

## Background

The centromere is the chromosomal locus responsible for the proper segregation of replicated sister chromatids to daughter cells during cell division. In all eukaryotes, the centromere is characterized by a unique chromatin structure that contains a centromere-specific histone 3 variant, called centromere protein (CENP)-A in mammals [1,2]. The kinetochore, a large multiprotein complex, is built onto this CENP-A chromatin and mediates microtubule attachment during mitosis and meiosis [3]. The CENP-A domain is flanked by heterochromatin, characterized by histone H3 methylated at lysine 9 (H3K9me), which may be important for centromeric chromatid cohesion, the last point of attachment between sister chromatids until the tightly coordinated metaphase to anaphase transition [4,5]. In addition, CENP-A domains are interspersed with domains containing histone H3 dimethylated at lysine 4 (H3K4me2), a modification associated with permissive chromatin [5-7].

Metazoan centromeres are generally composed of large amounts of highly repetitive 'satellite' DNA, which otherwise is remarkably unconserved in sequence. Human centromeres contain the 171 bp tandemly repeated alpha satellite DNA family, found in arrays of up to several megabase pairs at every endogenous centromere [8]. This large amount of highly homologous tandemly repeated DNA presents an obstacle against understanding the organization of chromatin domains at human centromeres.

Human neocentromeres are ectopic centromeres that have formed in non-centromeric locations and are devoid of alpha satellite DNA. Approximately 93 neocentromeres have been identified to date, mainly by clinical cytogenetic laboratories, because they lead to the mitotic stability of what would otherwise be an acentric chromosomal fragment. Although formation of neocentromeres has been found on 21 of the human chromosomes, certain regions appear to have a high propensity to form neocentromeres, such as chromosomes 3q, 15q, and especially 13q, of which 16 cases have been described [9,10]. However, CENP-A chromatin immunoprecipitation (ChIP) on CHIP (microarray) analysis of three neocentromeres cytologically localized to band 13q32 and two localized to band 13q21, demonstrated that each formed on a distinct genomic location with no detectable sequence similarity or tandemly repeated DNA [11,12]. This analysis demonstrated that neocentromeres are epigenetically determined, with little involvement of the primary DNA sequence. Neocentromeres have been induced experimentally in a variety of organisms, including *Schizosaccharomyces pombe*, *Candida albicans*, barley cultivars and *Drosophila *[13-16]. Both experimentally induced and clinical neocentromeres form on unique sequences and contain CENP-A, the epigenetic mark for centromere formation [1].

The formation of human neocentromeres on single copy DNA sequences presents an important opportunity to investigate centromeric chromatin domain structure in relation to the underlying DNA sequence. Higher-resolution ChIP on CHIP analysis of a neocentromere in band 13q32 showed precise colocalization of CENP-C and CENP-H with CENP-A, organized into distinct major and minor domains that defined a unique centromeric chromatin structure [17]. In this study, we investigated further the chromatin domain organization of three independent neocentromeres from chromosome 13q. Each of these neocentromeres displays a similar two-domain CENP-A organization. We observed additional CENP-A colocalizing with the 5' end of genes and with H3K4me2 in the vicinity of neocentromeres. Unexpectedly, we did not detect any neocentromere-specific H3K4me2 domain associated with the CENP-A domains. We also found a surprising paucity of heterochromatin near the CENP-A domains of these neocentromeres, which may explain a defect in centromere cohesion observed at the neocentromere. Thus, this study provides important insights into the structural and epigenetic requirements for centromere function.

## Results

### High-resolution analysis of chromosome 13q neocentromeres

**Figure 1:**
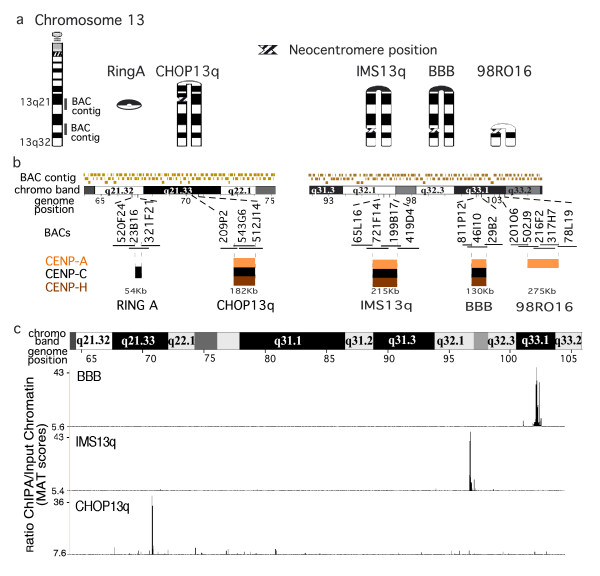
**Chromatin immunoprecipitation (ChIP)-CHIP (microarray) analysis of chromosome 13q neocentromeres**. (a) Ideograms of four inverted duplication 13q chromosomes, shown arched at the inversion breakpoint to indicate the duplicate regions homologous to (left) the normal chromosome 13. A ring chromosome derived from band 13q21 is also shown. The name of the cell line and the cytological position of each of the neocentromeres are indicated. The position of the 13q21 and 13q32 BAC microarrays are shown on the ideogram of the normal chromosome 13. (b) Expanded areas showing the 13q21 bacterial artificial chromosome (BAC) microarray (11 Mbp, 103 BACs)[12] and the 13q32 BAC microarray (14 Mbp, 126 BACs) [11]. The region that contained each neocentromere is expanded below, showing the BACs and their overlaps in each region. The positions of centromere protein (CENP)-A, -C and/or -H are shown when determined. The results showing colocalization of CENP-A, -C and -H on the IMS13q neocentromere are modified from a previous report [12]. (c) Affymetrix high density tiling array analysis of cell lines (CHOP13q, IMS13q and BBB), showing ~42 Mbp region encompassing the BAC microarrays and region between them on chromosome 13q21 to 13q33. The distinct and specific CENP-A domain identified for each neocentromere are shown. CENP-A chromatin immunoprecipitation (ChIP) model-based analysis of tiling-array (MAT) score: CHOP13q p < 10-^10^; IMS13q, *P *< 10-^8^; BBB, *P *< 10-^8^).

The genomic positions of five neocentromeres derived from chromosome 13q have been previously localized using ChIP with antibodies to inner kinetochore proteins hybridized to two custom bacterial artificial chromosome (BAC) microarrays from 13q32 and 13q21 (Figure 1a, b) [11,12]. These results demonstrated that at three neocentromeres, CENP-A, -C and -H precisely colocalized at the resolution of these BAC arrays. To further investigate the structure of these neocentromeres, we used the whole genome tiling arrays (Affymetrix, Santa Clara, CA, USA), which represent all nonrepetitive elements of chromosome 13 with ~35 bp resolution. CENP-A ChIP on CHIP revealed the position of each of the three neocentromeres in cell lines BBB, IMS13q and CHOP13q (Figure 1c), which were in agreement with the positions determined by the BAC microarrays. This analysis revealed a high degree of specificity for each neocentromere position in each cell line, with no significant CENP-A signal at the neocentromere position in the other cell lines.

**Figure 2:**
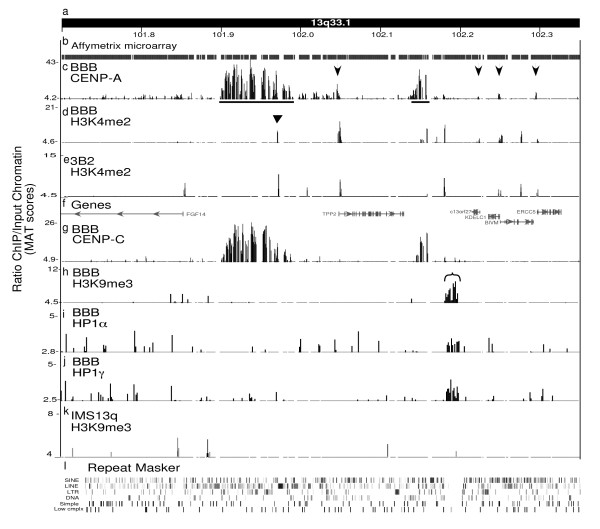
**High-resolution analysis of chromatin domains at the BBB neocentromere**. A 650 kb genomic region containing the neocentromere in cell line BBB, showing (a) the UCSC Hg18 genome coordinates from band 13q33.1 and (b) the coverage of the region on the Affymetrix tiling array, where gaps represent repetitive DNA not included on the array. (c) Results from chromatin immunoprecipitation (ChIP) with antibodies to centromere protein (CENP)-A from cell line BBB; model-based analysis of tiling-array (MAT) score. Horizontal lines indicate major and minor domains. Arrows indicate small domains that colocalize with the 5' end of the genes (see Figure 5). (d) ChIP with histone H3 dimethylated at lysine 4 (H3K4me2) antibody from cell line BBB;. Triangle indicates small domain within CENP-A domain. (e) ChIP with H3K4me2 antibody from fibroblast cell line 3B2, MAT *P *< 1.6 × 10-^5^, which serves as a non-neocentric control for BBB (f) The genes in the region. (g) ChIP with antibodies to CENP-C from cell line BBB. (h) ChIP with antibodies to H3K9me3 from cell line BBB. (i) ChIP with antibodies to heterochromatin protein (HP)1α from cell line BBB. (j) ChIP with antibodies to HP1γ from cell line BBB. (k) ChIP with antibodies to H3K9me3 from cell line IMS13q. (l) Repeat Masker tracks, where the 21.6 kb transposon-free region colocalizing with the H3K9me3 and HP1 domains can be seen. For domain coordinates and P values, see Table 1.

**Figure 3:**
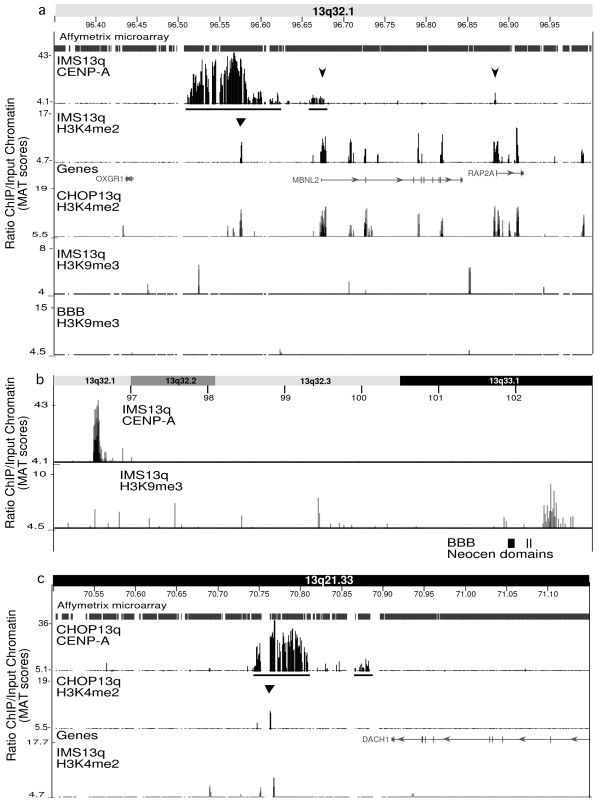
**High-resolution analysis of chromatin domains at neocentromeres from IMS13q and CHOP13q cell lines**. (a) A 650 kb genomic region surrounding the neocentromere in cell line IMS13q, showing the results for the chromatin immunoprecipitation (ChIP) analysis using antibodies to centromere protein (CENP)-A and H3K4me2. Arrows indicate small CENP-A domain colocalizing with the 5' end of genes (see Figure 5). Triangle indicates small histone H3 dimethylated at lysine 4 (H3K4me2) domain within the CENP-A domain. The H3K4me2 ChIP from CHOP13 serves as a control for IMS13q neocentromere region. H3K9me3 ChIP results for IMS13q and BBB are shown. (b) A 7 Mbp region containing the IMS13q CENP-A domain in band 13q32.1 and the H3K9me3 domain seen in this cell line in chromosome band 13q33.1. Note BBB CENP-A and H3K9me3 domain positions indicated (see Figure 2). (c) A 650 kb genomic region surrounding the neocentromere in cell line CHOP13q; ChIP for CENP-A and H3K4me2 is shown. The H3K4me2 ChIP from IMS13q serves as a control for CHOP13q neocentromere region. For domain coordinates and P values, see Table 1.

The Affymetrix CHIP data confirmed the major and minor domain structure of the kinetochore chromatin in the neocentromere from cell line BBB (Figure 2c). The domain sizes were adjusted somewhat from a previously published PCR microarray, due to the higher resolution and sensitivity of the Affymetrix CHIP [17]. Interestingly, the other two 13q neocentromeres in cell lines IMS13q and CHOP13q showed a similar major and minor CENP-A domain structure (Figure 3a, c). Major domains ranged from ~75 to ~90 kb in size, separated by intervening domains of ~60 to ~150 kb that were devoid of CENP-A, and minor domains of ~10 to ~20 kb in size (Figure 2c, Figure 3a, c). The UCSC-Hg18 genome coordinates for these domains were obtained using the model-based analysis of tiling-array (MAT) log score [18] at *P *values of 10-^5^, and are listed in Table 1. Consistent with our original findings [11,12,17], this high-resolution analysis revealed that these major and minor domains occur precisely between genes, most strikingly in the relatively gene-rich BBB neocentromere region (Figure 2f, Figure 3).

**Table 1:** Size and position of chromatin domains at neocentromeres.

Cell Line	Domain	Size (kb)	Coord (hg18) chromosome 13	P value
BBB	CENP-A major	89.7	101,901,496-101,991,165	<10^-5^*
BBB	CENP-A minor	19	102,140,584-102,159,277	
BBB	Intervening	142	101,998,994-102,140,584	
BBB	CENP-C major	89.7	101,901,496-101,991,165	<10^-5^
BBB	CENP-C minor	19	102,140,584-102,159,277	<10^-5^
BBB	H3K4me2	0.740	101,969,955-101,970,694	<10^-5^
BBB	H3K9me3	15	102,183,799-102,198,988	<2.5 × 10^-5^
BBB	HP1α	15	102,183,799-102,198,988	<5.6 × 10^-5^
BBB	HP1γ	15	102,183,799-102,198,988	<9.6 × 10^-5^
IMS13q	CENP-A major	90	96,511,130-96,601,103	<10^-5^
IMS13q	CENP-A minor	15	96,661,230-96,676,244	
IMS13q	Intervening	60	96,601,103-96,661,230	
IMS13q	H3K4me2	1.8	96,575,232-96,577,051	<10^-5^
IMS13q	H3K9me3	159.5	102,375,370-102,531,950	<10^-5^
CHOP13q	CENP-A major	74	70,735,337-70,809,172	<10^-5^
CHOP13q	CENP-A minor	10	70,872,442-70,883,219	
CHOP13q	intervening	64	70,809,175-70,872,440	
CHOP13q	H3K4me2	0.7	70,763,283-70,763,914	<10^-5^

*CENP-A major, minor and intervening domains are from same CENP-A ChIP for each cell line, and therefore are determined using same p value.

Additional weak but significant CENP-A signals in the vicinity of neocentromeres were observed at the 5' end of genes (Figure 2c, Figure 3a, arrowed). In the BBB neocentromere, four significant CENP-A domains precisely colocalized with the 5' end of the genes in this region (Figure 2c). In the IMS13q neocentromere, two distinct CENP-A domains were observed at the 5' end of genes, including the distal end of the minor domain (Figure 3a). Notably, the CHOP13q neocentromere region does not contain the 5' ends of genes, and additional domains of CENP-A were not observed (Figure 3c). Importantly, these extra CENP-A peaks did not correlate with the CENP-C peaks in the region (Figure 2g), suggesting that they are not involved in the kinetochore structure.

**Figure 4:**
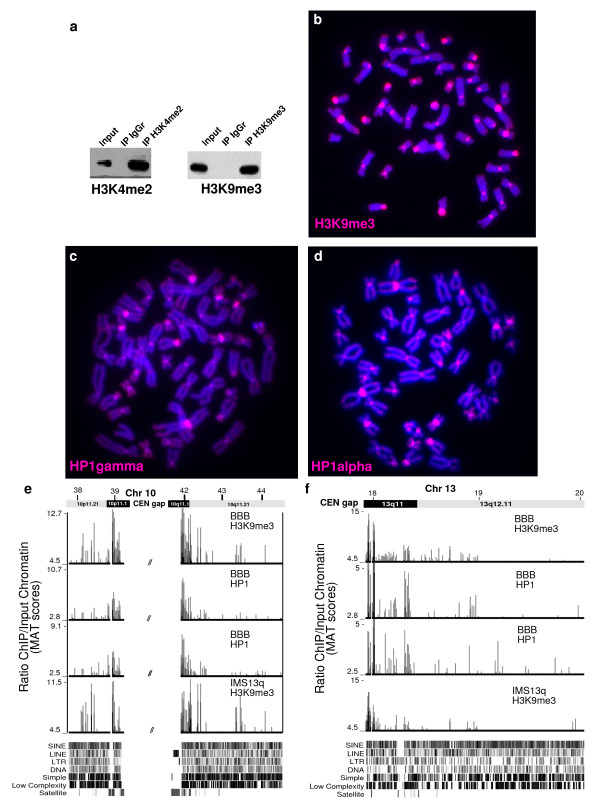
**ChIP analysis at endogenous centromeres**. (a) Western blot analysis of histone H3 dimethylated at lysine 4 (H3K4me2) and histone H3 methylated at lysine 9 (H3K9me) chromatin immunoprecipitation (ChIP). Lanes-Input chromatin, ChIP with rabbit IgG, and ChIP with antibody to (left) H3K4me2 or (right) H3K9me3. (b, c, d) DNA from the each ChIP experiment from the indicated antibody was labeled and used as a probe on metaphase spreads. H3K9me3, HP1α and HP1γ ChIP DNA hybridized to centromeric/pericentromeric regions. (e) An area of ~6 Mbp around the centromere gap of chromosome 10. (f) An area of ~2 Mbp at the centromere of chromosome 13. ChIP on CHIP (array) results for indicated antibodies and cell lines. All antibodies shown are enriched at the pericentromeric region. In chromosome 10, the pericentric heterochromatin is present over ~1 Mb at each side of the centromere gap. In chromosome 13, heterochromatin extends approximately 0.5 Mbp on the q arm. Note that genomic satellite DNA is not included on the Affymetrix CHIP. H3K9me3 (BBB) MAT score *P *< 2.5 × 10-^5^, HP1α (BBB) *P *< 5.6 × 10-^3 ^and HP1γ (BBB) *P *< 9.6 × 10-^3^, H3K9me3 (IMS13q) *P *< 1.6 × 10-^5^. Repeat Masker tracks are shown below the graphs.

H3K4me2 was reported to be interspersed with CENP-A domains on alpha satellite DNA at endogenous centromeres, defining a distinct centrochromatin that may play a structural role [5]. Therefore, the organization of H3K4me2 was investigated at the three neocentromeres. The ChIP analysis of H3K4me2 was validated by western blotting (Figure 4a) and the association with the 5' end of genes across the entire chromosome 13, as previously reported (see Additional file 1) [7]. H3K4me2 was also found on the 5' end of genes in the vicinity of the neocentromeres that also bound CENP-A (Figure 2d, e, Figure 3a, c). In addition, we observed within each of the major CENP-A domains an additional small domain (~700 to 1500 bp) of H3K4me2 that did not colocalize with a gene (Table 1) (Figure 2e and Figure 3a, c, triangle). However, these domains of H3K4me2 were also observed in the same locations in control cell lines (Figure 2e, Figure 3a, c) and thus are not specific to the neocentromeres. These results suggest that H3K4me2 is not a predominant marker interspersed with the CENP-A chromatin at neocentromeres.

**Figure 5:**
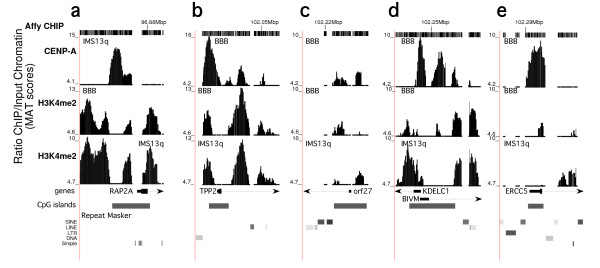
**High-resolution analysis of centromere protein (CENP)-A and histone H3 dimethylated at lysine 4 (H3K4me2) at promoters in the vicinity of neocentromeres**. Area of (a) ~4 kb at the promoter region of the *RAP2A *gene near the neocentromere in IMS13q (see Figure 3a); (b) ~4.8 kb at the promoter of the *TPP2 *gene in BBB (see Figure 2); (c) ~1500 bp at the *c13orf *gene in BBB; (d) ~4 kb at the *KdelC1 *and *BIVM *genes in BBB. (e) ~2200 bp at the *ERCC5 *gene in BBB. For each panel (a-e), the neocentric CENP-A domain is shown, and the H3K4me2 domains for both BBB and IMS13q lines. The transcription start site and CpG island for each gene are indicated. In general, the CENP-A is found in a distinct location from the H3K4me2 and appears to favor CpG islands.

Higher-resolution analysis of the CENP-A and H3K4me2 domains at the 5' end of the genes around the neocentromeres revealed that in general, CENP-A and H3K4me2 do not precisely overlap but instead appear to occupy distinct locations in these regions. Analysis of the promoter regions of these genes showed that the CENP-A domains roughly correlate with CpG islands in the regions, whereas H3K4me2 appears to flank these CpG islands (Figure 5). In some regions, both CENP-A and H3K4me2 appear to colocalize, which may represent occupation on neighboring nucleosomes or differences within the cell population. Notably, the major and minor CENP-A domains at the neocentromeres do not contain CpG islands, thus CpG islands do not appear to be specifying neocentric CENP-A domains.

### Heterochromatin at neocentromeres

A large heterochromatin domain appears to be a ubiquitous feature of metazoan centromeres [2] involved in retention of centromeric sister chromatid cohesion [4,19,20]. Previously Suv39H1, the histone H3K9 methylase, was specifically found at the BBB neocentromere, but not on the corresponding genomic regions on the other chromosome 13 areas [21]. Therefore, the size and extent of the centromeric heterochromatin, characterized by H3K9me3 and heterochromatin protein 1 (HP1) was evaluated at the BBB neocentromere. ChIP using antibodies to H3K9me3, HP1α and HP1γ was validated by fluorescent *in situ *hybridization (FISH) (Figure 4b, c, d) and by the enrichment of endogenous pericentric heterochromatin sequences present in the Affymetrix tiling array (Figure 4e, f). At the BBB neocentromere, ChIP with H3K9me3 revealed a 15 kb chromatin domain containing this modification about 15 kb distal to the minor CENP-A domain (Table 1). Further ChIP analysis showed colocalization with both HP1α and HP1γ, suggesting that this is a bona fide heterochromatin domain (Figure 2h, i, j). Analysis of the genomic sequence in this heterochromatin region revealed an unexpected location within a 21.6 kb region that is completely free of transposable elements. There are no additional repeat elements or genes within this region. Analysis of 26 previously described transposon-free regions of >10 kb on chromosome 13 [22] revealed that only three contained detectable H3K9me3 (data not shown), showing that heterochromatin formation is not a general property of these regions.

Further examination of H3K9me3 at the IMS13q neocentromere revealed no significant signal for heterochromatin near this neocentromere. Indeed, in this cell line, the closest significant block of H3K9me3 was found several mega base pairs distant in band 13q33.1 (distal to the location of the BBB neocentromere) (Table 1) (Figure 3b), which was the only block of heterochromatin detected on the long arm of chromosome 13 in this cell line. Note that in the IMS13q cell line, the small block of heterochromatin observed in the BBB neocentromere was not present (Figure 2k).

**Figure 6:**
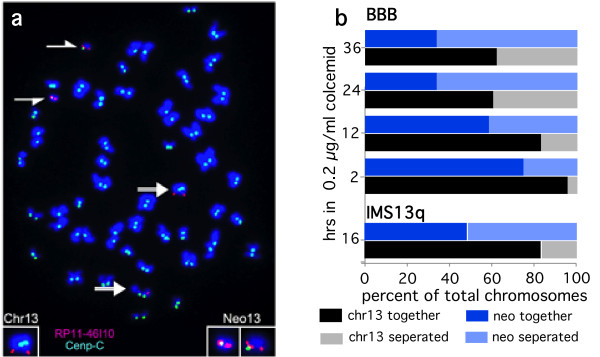
**Premature separation of neocentric metaphase chromosomes**. (a) BBB cells treated with colcemid for ~24 hours show highly condensed chromosomes with separated arms but attached centromeres, as indicated by immunofluorescence with anti-centromere protein (CENP)-C (green). Fluorescent *in situ *hybridization (FISH) probe RP11-46I10 (red) from band 13q32 is found on the q arm of the two normal chromosomes 13 (full arrows) and on both ends of symmetric invdup13q neocentromere chromosome (see Fig 1). The neocentric chromosome has separated into its two replicated chromatids (split arrows), each of which contains a single kinetochore (green) which colocalizes with one of the RP11-46I10 FISH signals. (b) Quantification of separation of normal chromosome 13 (black and gray) and invdup13q neocentric (dark and light blue) chromosome at various times in colcemid. IMS13q at 16hours: 8/52 normal separated, 26/52 neocentric separated. BBB at 2 hours: 4/92 (4.5%) normal separated, 15/51 (30%) neocentric separated; at 12 hours: 17/103 (17%) normal separated, 26/57 (46%) neocentric separated; at 24 hours: 140/358 (39%) normal separated, 145/210 (70%) neocentric separated; at 36 hours: 41/108 (38%) normal separated, 39/56 (70%) neocentric separated.

The surprising paucity of heterochromatin in these neocentromeres prompted us to examine whether they displayed any defects in centromeric sister chromatid cohesion. Upon prolonged exposure to microtubule depolymerizing drugs, centromeres remain attached after chromatid arms fully separated [23]. We therefore treated actively growing BBB and IMS13q cells with colcemid, and quantified the attached versus separated centromeres in the normal chromosome 13 and invdup13q neocentric chromosomes, using immunofluorescence and FISH (Figure 6a). In the BBB cell line, after 2 hours, 4.5% of chromosomes 13 were separated compared with 30% of the neocentric chromosomes. This difference increased over time: by 24 and 36 hours of colcemid treatment, 70% of neocentric chromosomes were separated compared with only 38% of the normal chromosome 13 (Figure 6b). In IMS13q, after 16 hours in colcemid, 50% of neocentromeres were separated compared with only 15% of normal chromosomes 13. Thus, under these conditions these neocentric chromosomes display a premature separation phenotype.

## Discussion

This report describes the organization of chromatin domains across a human centromere, taking advantage of the fact that neocentromere formation on single-copy DNA permits mapping across the region using the ChIP on CHIP method. A two-domain CENP-A chromatin domain structure was observed at three neocentromeres, with each displaying major and minor domains (Figure 2c, Figure 3a, c). This organization may suggest a chromatin loop model, as proposed for endogenous centromeres [2,24]. The organization of these domains suggests a single chromatin loop, with the two CENP-A domains juxtaposed and forming a surface for kinetochore formation. Such models can be addressed using chromosome conformation capture methods [25] at neocentromeres because of the sequence complexity of the genomic region.

The location of the CENP-A domains at these neocentromeres suggests that they form predominantly in gene-poor regions and between genes. At both the BBB and IMS13q neocentromeres, both the major and minor domains fall precisely between genes (Figure 2, Figure 3a). At the CHOP13q neocentromere, the CENP-A domain borders the 3' end of a gene (Figure 3c). A previously mapped neocentromere (98RO16) occurs in a gene desert of ~2.5 Mbp (Figure 1b) [11]. The fact that human chromosome 13 is relatively gene-poor might provide more opportunity for neocentromere formation, which may partially explain the disproportionate number of neocentromeres seen on this chromosome [10,26]. Our data are consistent with the intergenic locations of endogenous CENP-A domains at rice centromeres [27], the locations of evolutionary new centromeres in gene deserts [28], and experimentally induced neocentromeres in *C. albicans *[14]. By contrast, in both a human neocentromere and an artificially generated centromere, gene expression was observed despite the presence of CENP-A [29,30].

Unlike at endogenous human centromeres [5,6], a large domain of centrochromatin, defined as interspersed domains of CENP-A and H3K4me2, was not observed at neocentromeres (Figure 2, 3). Small H3K4me2 domains were observed within the major CENP-A domain, but these were also observed in non-neocentric chromosomes. Thus, the minimal amount of H3K4me2 and its lack of specificity at the neocentromere suggest that it is unlikely to play a significant higher-order structural role in kinetochore formation at neocentromeres. Instead, H3K4me2 was seen across the chromosome associated with the 5' end of genes, as expected [7].

CENP-A was found in the promoters of genes near the neocentromere, which correlated with CpG islands (Figure 5). A previous study showed localization of the budding yeast homolog of CENP-A, Cse4, with promoters of the most strongly expressed genes throughout the genome [31]. CENP-A has been associated with chromatin remodeler components such as RSF (remodeling and spacing factor) and RbAp46/48 [32,33], which are also known to act at gene promoters. It seems possible that CENP-A incorporation into neocentromeres by chromatin remodelers may also lead to localized incorporation into the promoters of nearby genes, which are regions of high nucleosomal turnover. At endogenous centromeres, the absence of nearby gene promoters and the abundance of heterochromatin would eliminate this possibility.

The remarkable paucity of heterochromatin observed at the BBB and IMS13q neocentromere was unexpected at a functional human centromere (Figure 2, 3). All other metazoan centromeres described to date contain significant amounts of heterochromatin, as indicated by H3K9me and HP1 [5,34], which may be important for centromeric sister chromatid cohesion. The presence of this heterochromatin domain in a distinct transposon-free region in BBB may suggest a chromatin and/or a DNA sequence bias favorable to heterochromatin formation. However, this heterochromatin domain is not seen in IMS13q (Figure 2k), which acts as a control, although cell type differences may explain these epigenetic changes (BBB is a transformed fibroblast line and IMS13q is a lymphoblast line). Nevertheless, there is no significant heterochromatin domain near the IMS13q neocentromere, the closest one being ~5 Mbp distant, distal to the BBB neocentromere (this domain is not observed in the BBB cell line) (Figure 3a, b). Differences between these rare neocentromere cell lines make it difficult to tell if the neocentromeres play a role in inducing these domains of heterochromatin.

The paucity of heterochromatin domain at these neocentromeres is consistent with the observation that the neocentric sister chromatids separate prematurely compared with the endogenous chromosomes (Figure 6). Thus, it appears that centromeric cohesion may not be as well established at the neocentromere and that these chromosomes may rely more strongly on arm cohesion for segregation. However, given the high degree of stability of this neocentric chromosome in normally cycling cells [9], this defect may only manifest itself when the spindle assembly checkpoint is activated. It will be of great interest to determine the presence and location of components of centromeric cohesion such as cohesin and shugoshin on the neocentromere [35].

These neocentromeres have been shown to have neither specific centromeric sequences nor significant heterochromatin, and to occur near genes. Neocentromere formation has been proposed to be the crucial first step in the seeding of an evolutionarily new centromere, which then becomes fixed in a species, resulting in a centromere repositioning event [28,36]. This study reveals that these neocentromeres can indeed start off with minimal to no heterochromatin structure and still be functional. Fixation of these neocentromeres in a species is accompanied by an expansion of centromeric sequences and heterochromatin at the new centromere [37], which may be required for increased mitotic stability or for insulation from genes.

## Conclusions

In this report, three human neocentromeres were analysed with high-resolution chromatin immunoprecipitation (ChIP) on CHIP (microarray) in order to investigate centromeric chromatin domain structure. Each neocentromere contained ~100 kb of centromere protein (CENP)-A in a two-domain organization, with additional CENP-A domains coinciding with CpG islands at the 5' end of genes in the vicinity of neocentromeres. Analysis of histone H3 dimethylated at lysine 4 (H3K4me2) revealed small domains at each neocentromere, suggesting that this mark does not play a higher-order structural role at neocentromeres as has been proposed for endogenous centromeres [5]. A surprising paucity of heterochromatin was observed at these neocentromeres, suggesting that large domains of heterochromatin are not strictly required for centromere function. However, a defect in centromere cohesion at these neocentromeres may be attributable to the paucity of heterochromatin domains. Thus, this study provides important insight into the structural requirements for human centromere function.

## Methods

### Antibodies

ChIP-Grade antibodies were mouse monoclonal anti-CENP-A, rabbit polyclonal anti-histone H3 (dimethyl K4) and anti-histone H3 (trimethyl K9) (both from Abcam Inc. Cambridge, MA, USA), and mouse monoclonals anti-HP1α and anti-HP1γ (both from Millipore Corp., Temecula, CA, USA). Rabbit polyclonal anti CENP-C was a gift (Bill Earnshaw, Institute of Molecular and Cell Biology, Edinburgh, UK). Mouse and rabbit IgG were also used (Vector Laboratories, Burlingame, CA, USA).

### Chromatin immunoprecipitation assays

Epstein-Barr virus-transformed lymphoblast lines IMS13q and CHOP13q and fibroblast BBB were grown in standard media. Immunoprecipitation from soluble chromatin obtained by microccocal nuclease digestion to mononucleosomes was performed as previously described [11]. Immunoprecipitation from cross-linked, sonicated extracts were performed as previously described [17], with the following modification: cross-linked extracts were sonicated using a 10 minute pulse (high setting, 30 seconds on, 30 seconds off) (Bioruptor UCD-200 sonicator; Diagenode Inc., Sparta, USA), to obtain a 200-400 bp ladder. Aliquotes (25 to 30 μg) of chromatin were immunoprecipitated with 4 to 8 μg of antibody in accordance with the manufacturer's instructions.

### PCR amplification, labeling of chromatin DNA and microarray hybridization

Between 10 and 20 ng of immunoprecipitated or input DNA were end-repaired and amplified by PCR as previously described [17]. Aliquots (9 μg) of the amplified DNA were fragmented and biotin-labeled (GeneChip^® ^WT Double-stranded DNA Terminal Labelling Kit; Affymetrix, Santa Clara, CA, USA). Input and immunoprecipitated labeled DNA were hybridized to a micorarray chip (GeneChip^® ^Human Tiling 2.0R G Array; Affymetrix), which includes chromosomes 10, 13, 14 and 17. The chips were washed and stained (GeneChip^® ^Fluidics Station 450; Affymetrix), then scanned (GeneChip^® ^Scanner 3000 7G and GeneChip^® ^Operating Software (GCOS); Affymetrix).

### Microarray analysis and Statistical analysis

Files generated by GCOS (cel files) were analyzed (Tiling Analysis Software (TAS) V.1.1; Affymetrix) and displayed in the Integrated Genome Browser http://genoviz.sourceforge.net/. Duplicate experiments were processed using the MAT algorithm [18] normalizing ChIP and input signal (cel files). The normalized MAT score values were displayed in the UCSC genome browser Hg18 http://genome.ucsc.edu/, using a *P*-value significance as a cut-off point. Raw data can be obtained at ArrayExpress under the accession number E-TABM-705. The normalized data can be obtained from the ftp site of the same experiment.

### Cell arrest and mitotic shake

BBB cells (3 × 10^6^) were plated in T175 flasks 24 hrs before cell cycle arrest. Colcemid 0.2 μg/ml (Roche, IN, USA) was added for 2, 12, 24 and 36 hours, after which mitotic cells were collected by a shake and incubated for 14 min at 37°C in a 25 mM KCl/0.27% Na citrate hypotonic solution. and Immunofluorescence on 3:1 methanol:acetic acid-fixed chromosomes were performed as described [9]. Images were collected using microscope (E800; Nikon, Tokyo Japan) connected to a digital camera (DKC 5000; Sony, Tokyo, Japan), using single-pass filters for fluorescein isothiocyanate, tetramethyl rhodamine isothiocyanate and 4',6-diamidino-2-phenylindole (DAPI) (Chroma, Brattleboro, VT, USA) and merged using Adobe Photoshop software (Adobe Systems Inc., San Jose, CA, USA).

## Competing interests

The authors declare that they have no competing interests.

## Authors' contributions

AA, DH and FC performed experiments, AA, DH and PEW designed experiments, AA and PEW wrote the paper. All authors read and approved the final manuscript.

## Supplementary Material

Additional file 1 **H3K4me2 is present at the 5' end of genes**. **(a) **An ideogram of chromosome 13 is shown. The expanded area below it indicates the extent of the Affymetrix CHIP coverage on this chromosome (positions 20-114 Mbp). Results from BBB cell extracts precipitated by chromatin immunoprecipitation (ChIP) with antibodies to H3K4me2 are shown, model-based analysis of tiling-array (MAT) score *P *< 10-^5^. Genes are indicated below the graph. Note the strong correlation between histone H3 dimethylated at lysine 4 (H3K4me2) and genes and its absence in gene desert regions. **(b-e)**. Close-up view of the 5' end of four different genes. **(e) **An area of 10 kb of the 5' end of the *DACH1 *gene, whose 3' end domain is 27 kb distal to the minor centromere protein (CENP)-A domain in CHOP13q. Click here for file
