# Supplementary figures and images for: A paucity of heterochromatin at functional human neocentromeres
Source: Epigenetics Chromatin. 2010 Mar 8;3:6. doi: 10.1186/1756-8935-3-6 (PMC2845132; doi:10.1186/1756-8935-3-6)

# Supplementary Figure 1, Alonso et al.

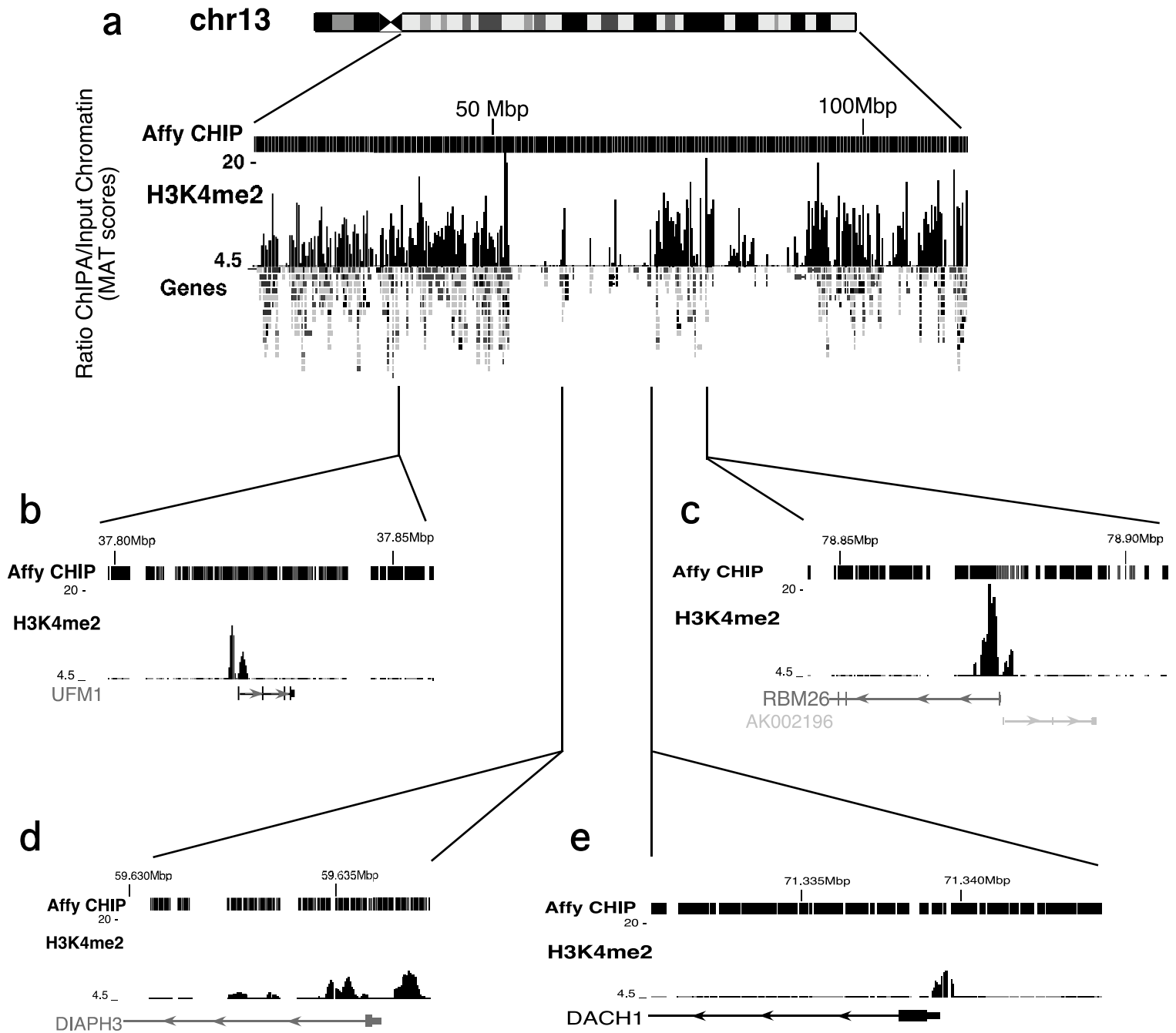

Supplement: Additional file 1 — H3K4me2 is present at the 5' end of genes. (a) An ideogram of chromosome 13 is shown. The expanded area below it indicates the extent of the Affymetrix CHIP coverage on this chromosome (positions 20-114 Mbp). Results from BBB cell extracts precipitated by chromatin immunoprecipitation (ChIP) with antibodies to H3K4me2 are shown, model-based analysis of tiling-array (MAT) score P < 10-5. Genes are indicated below the graph. Note the strong correlation between histone H3 dimethylated at lysine 4 (H3K4me2) and genes and its absence in gene desert regions. (b-e). Close-up view of the 5' end of four different genes. (e) An area of 10 kb of the 5' end of the DACH1 gene, whose 3' end domain is 27 kb distal to the minor centromere protein (CENP)-A domain in CHOP13q. [file 1756-8935-3-6-S1.PDF]
